# Total Flavonoids from Radix Glycyrrhiza Exert Anti-Inflammatory and Antitumorigenic Effects by Inactivating iNOS Signaling Pathways

**DOI:** 10.1155/2018/6714282

**Published:** 2018-05-22

**Authors:** Yi-Xin Jiang, Yang-Yi Dai, Yi-Feng Pan, Xi-Min Wu, Yue Yang, Ka Bian, Dan-Dan Zhang

**Affiliations:** ^1^Shanghai University of Traditional Chinese Medicine, Shanghai 201203, China; ^2^Shanghai Huanghai Pharmaceutical Co. LTD., Shanghai 200051, China; ^3^Department of Biochemistry and Molecular Medicine, George Washington University, Washington, DC 20052, USA; ^4^George Washington Cancer Center, Washington, DC 20052, USA

## Abstract

Inducible nitric oxide synthase (iNOS) plays an important role in inflammation, which has also been considered as a major driver of breast cancer disease progression. Radix Glycyrrhiza (RG) has been broadly used for its anti-inflammatory and antitumorigenic effects. However, the mechanisms of regulation of iNOS in inflammation and cancer have not been fully explored. Total flavonoids isolated from RG (TFRG) exhibited anti-inflammatory activity through the regulation of ERK/NF-*κ*B/miR-155 signaling and suppression of iNOS expression in LPS/IFN-*γ* stimulated RAW264.7 macrophages without cytotoxicity. TFRG also markedly reduced tumor mass of breast cancer cell MDA-MB-231 xenografts with suppression of iNOS expression, formation of 3-nitrotyrosine (3-NT), and inactivation of protumorigenic JAK2/STAT3 signaling pathway. These results suggested that TFRG limited the development of breast cancer and inflammation due to its property of iNOS inhibition.

## 1. Introduction

The linkage between chronic inflammation and tumor development has been studied for decades [1, 2]. The chronic inflammation plays an important role in the pathogenesis of various cancers including breast cancer [3, 4]. Inducible nitric oxide synthase (iNOS), one of three enzyme isoforms of nitric oxide synthase (NOS), is the key enzyme expressed under inflammatory conditions and capable of producing high levels of nitric oxide (NO), which impact the redox state of cells and induce oxidation of proteins, lipids, and DNA. For instance, NO-derived reactive nitrogen species (RNS) such as peroxynitrite (ONOO^−^) can S-nitrosylate COX-2, leading to the increase in its proinflammatory activity [5]. In estrogen receptor- (ER-) negative breast cancer patients, the iNOS expression is positively correlated with tumor-node metastasis (TNM), a marker clinically used to predict patient survival [6]. iNOS is also closely associate with many aggressive tumor phenotypes and treatment-resistant cancers [7]. Several iNOS inhibitors have demonstrated efficacy in reduction of metastases and inhibition of cancer stem cell renewal in triple-negative breast cancer [8]. Previous research has demonstrated inhibitory effect of iNOS blockage on tumor growth and metastases [9]. It is obvious that controlling iNOS-mediated inflammation can serve as a therapeutic strategy to inhibit tumor progression and metastasis [10].

We hypothesize that plant extracts with anti-inflammatory capability targeting iNOS and oxidative/nitrosative stress are useful chemopreventive agents against breast cancer growth and/or metastasis.

Radix Glycyrrhiza (RG), the root of* Glycyrrhiza* species (Leguminosae), has been broadly used in various Traditional Chinese Medicine (TCM) formulas. RG possesses a wide range of pharmacological effects including antiviral [11], anti-inflammatory [12, 13], antioxidant [14], and antiobesity activities [15]. RG has also been reported to have potent immune modulating capability [16, 17]. Additionally, RG is commonly used as an additive in drug, food, and beverage preparations for its sweet taste. Meanwhile, its safeness has been well documented through numerous studies [18, 19].

Our previous study revealed that 70% ethanol extract from RG inhibited over 50% production of NO in LPS/IFN*γ*-costimulated RAW246.7 cells without eliciting significant cytotoxicity [20]. In this study, we report that total flavonoids isolated from RG (TFRG) exhibited anti-inflammatory activity through the regulation of ERK/NF-*κ*B/miR-155 signaling and suppression of iNOS expression. TFRG also markedly reduced tumor mass of MDA-MB-231 xenografts with inhibition of iNOS and formation of nitrotyrosine by JAK2/STAT3 signaling pathway.

## 2. Materials and Methods

### 2.1. Extract and Fractions Preparation

RG was purchased from Yang He Tang Company (Zhangjiang High-Tech Park, Shanghai, China) and affirmed by Shanghai Institute for Food and Drug Control (SIFDC). Dried RG was refluxed with 10x volume of 70% ethanol for 2 h three times, and the filtrate was concentrated to dryness under reduced pressure by a rotary evaporator. The ethanol extract (EE) was suspended in distilled water and successively partitioned with petroleum ether, ethyl acetate, and* n*-butanol. Each of these organic solvent fractions including the petroleum ether (PEF), ethyl acetate (EEF),* n*-butanol (BF), and the remaining aqueous fraction (AF) was collected and concentrated. The ethyl acetate fraction was further subjected to open column chromatography with macroporous resin AB-8 [3.8 × 50 cm, ethanol-water = 0%, 10%, 30%, 50%, 70%] to generate five fractions. The subfraction of 70% ethanol solution was collected and concentrated as TFRG. TFRG were dissolved in DMSO and stored at −20°C until use. The final concentration of DMSO in the cell culture studies was controlled at 0.1% or less.

### 2.2. Determination of Total Flavonoids Content

Ultraviolet spectrophotometry (US) assay was used for quantitative determination of flavonoids content as previously described with some modifications. Briefly, extract was diluted with 30% ethanol to 0.1 mg/mL and absorption was measured at 334 nm after the addition of 1 mL of 1% potassium hydroxide for 15 min. The absorption was compared to that of a standard curve of liquiritin which is also diluted in 30% ethanol. The flavonoids content was calculated as liquiritin equivalent using the regression equation between standard quantity and absorbance.

### 2.3. Ultraperformance Liquid Chromatography Assay (UPLC)

TFRG were subjected to UPLC using Agilent-1100 (Agilent Technologies, Santa Clara, CA, USA) with C18 column (spec: 3.5 *μ*m, 4.6 × 150 mm; Waters). The mobile phase consisted of gradient mixture of CH_3_CN (solvent A) and 50 mM NH_4_OAc buffer containing 0.1% formic acid (solvent B), which were separately filtered through filter and degassed by sonicating for 3 min. Chromatographic condition was as follows: 0–30 min, 10%–30% A (v/v); 30–35 min, 30%–95% A (v/v). The flow rate of mobile phase was maintained at 0.8 ml/min throughout the analysis and detector wavelength was kept at 254 nm. Six reference standards (Isoliquiritin, Isoliquiritinapioside, Glycyrrhetinic acid, Liquiritinapioside, Isoliquiritigenin, and Liquiritin) were used.

### 2.4. Reagents

Isoliquiritin, Isoliquiritinapioside, Glycyrrhetinic acid, Liquiritinapioside, Isoliquiritigenin, and Liquiritin (all of purity above 98% by HPLC) were purchased from Tauto Biotech (Shanghai, China). Recombinant mouse IFN-*γ* was purchased from R&D Systems (Minneapolis, MN, USA). Lipopolysaccharide (from* Escherichia coli* 0111:B4 *γ*-irradiated), 3-(4,5-dimethylthiazol-2-yl)-2,5- diphenyltetrazolium (MTT), L-N6-(1-Iminoethyl)lysine hydrochloride (L-NIL), and Matrigel were purchased from Sigma (St Louis, MO, USA). ELISA for NF-*κ*B p50/p65 EZ-TFA transcription factor assay kit was purchased from Millipore (Bedford, MA, USA). 3-Nitrotyrosine ELISA Kit was purchased from Abcam (Cambridge, MA, USA). Antibodies used in this study include murine iNOS monoclonal antibody (BD Transduction Laboratories, Lexington, KY), GAPDH polyclonal antibody (Santa Cruz Biotechnology, Santa Cruz, CA), phosphor-p38, p38, phosphor-JNK, JNK, phosphor-ERK1/2 and ERK1/2, phosphor-JAK2, JAK2, phosphor-STAT3, and STAT3 polyclonal antibodies (Cell Signaling Technology, Beverly, MA, USA). RPMI-1640 was purchased from Gibco Invitrogen Corporation (Grand Island, NY, USA). Fetal bovine serum (FBS) was purchased from Hyclone (Logan, UT, USA); TRIzol Reagent, Reverse Transcription Kit, and SYBR Green PCR Master Mix regents were obtained from Invitrogen (Carlsbad, CA, USA).

### 2.5. Cell Culture

RAW264.7 and MDA-MB-231 cells were obtained from the American Type Culture Collection (Manassas, VA, USA). RAW264.7 cells were maintained in endotoxin-free RPMI1640 medium supplemented with 10% fetal bovine serum while MDA-MB-231 cells were maintained in Dulbecco's modification of Eagle's medium (DMEM) supplemented with 10% FBS under the condition at 37°C and 5% CO_2_.

### 2.6. Orthotopic Xenograft of MDA-MB-231 Cells

Female athymic nu/nu mice (4 weeks old) were obtained from the breeding section of Shanghai University of Traditional Chinese Medicine. All procedures performed in animal studies were conducted in accordance with approved Institutional Animal Care and Use Committee protocols and Guide for the Care and Use of Laboratory Animals of Shanghai University of TCM. MDA-MB-231 cells were resuspended in mixture of serum free medium :  Matrigel (1 : 100) at 2 × 10^7^ cells/ml and 200 *μ*l of this cell suspension was injected into 2nd mammary fat pads of mice. Two weeks after cell injection, mice were randomly assigned to the following groups (6 mice/group): control (vehicle, ig), TFRG (20, 100 mg/kg/d, ig), and L-NIL (10 mg/kg/d, ig). After 4 weeks, mice were sacrificed. Tumors were snap-frozen in liquid nitrogen and stored at −80°C.

### 2.7. Cell Viability Assay

Cell viability was measured using MTT assay. Briefly, cells were treated with TFRG at various concentrations in 96-well plates for 24 h at final concentration of DMSO that never exceeded 0.1%. The cells were incubated with 10 *μ*l MTT (5 mg/ml in phosphate-buffered saline, pH = 7.4) for 4 h at 37°C. Reactions were read at 490 nm using a microplate reader. Data were presented as viability (%) = [(OD of treated groups)/(OD of untreated group)] × 100.

### 2.8. Measurement of Nitrite and 3-Nitrotyrosine Contents

Level of nitrite in culture media was measured by Griess reaction and used as the indicator of iNOS activity and NO production. Briefly, RAW264.7 cells were treated with different concentrations of crude extract, fractions, and TFRG. L-NIL (50 *μ*M) was included as a positive control followed by stimulation of 100 ng/ml LPS plus 10 U/ml IFN-*γ* in 96-well plates. Supernatant was mixed with the same volume of Griess reagent (1% sulfanilamide, 0.1% naphthylethylenediamine dihydrochloride, and 5% phosphoric acid) and incubated at room temperature for 10 min. The reaction was read at 540 nM using a microplate reader (Molecular Devices, Sunnyvale, CA, USA) to obtain the amount of nitrite against a standard curve generated with sodium nitrite.

3-Nitrotyrosine (3-NT) is a relatively specific marker of RNS damage caused by peroxynitrite which derives from excessive NO and oxygen ions. The content of 3-NT in the tumors was quantified by 3-NT ELISA Kit according to manufacturer's protocol.

### 2.9. Nuclear Extract Preparation and NF-*κ*B Activity Assay

Nuclear extracts were obtained and NF-*κ*B activity was assessed using NF-*κ*B EZ-TFA transcription factor assay kit which detects the amount of p50/p65 in the nucleus. Briefly, nuclear extract was mixed with biotinylated oligonucleotide containing NF-*κ*B consensus sequence (5′-GGGACTTTCC-3′) and then transferred to a streptavidin-coated 96-well plate for 1 hat room temperature. After three washes, plates were incubated with anti-p65 or p50 antibody followed by the incubation with HRP-conjugated secondary antibody. The amount of p65 or p50 was visualized by the addition of tetramethylbenzidine (TMB) and measured at 450 nm.

### 2.10. qRT-PCR

Total RNA was extracted from cells or tissues using TRIzol regents according to the manufacturer's protocol: for qRT-PCR, mouse iNOS primer sense: 3′-GGAGCGAGTTGTGGATTGTC-5′, antisense: 3′-GTGAGGGCTTGGCTGAGTGAG-5′; GAPDH primer sense: 3′-GCTACAGCTTCACCACCACAG-5′, antisense: 3′-GGTCTTTACGGATGTCAACGTC-5′. And level of GAPDH mRNA was used as an internal standard for iNOS expression and ABI Prism 7000 was used to carry out the reactions.

### 2.11. TaqMan® MicroRNA Real-Time RT-PCR Assays

The reactions were set according to the manufacturer's protocol. Briefly, total RNA was purified by TRIzol. For each reaction, 10 ng of total RNA was used for reverse transcription using TaqMan MicroRNA Reverse Transcription Kit and reverse transcription primers for mmu-miR-155 and the housekeeping gene RNU6B. Real-time PCR quantification was performed using TaqMan PCR primers and TaqMan Universal Master Mix using the following conditions: 16°C for 30 min, 42°C for 30 min, and 85°C for 5 min on Applied Biosystems 7500HT Fast Real-Time PCR System. The samples were measured in triplicate cases. RNU6B endogenous control was used for normalization, and expression levels were presented as 2^−ΔΔCT^ with standard deviation.

### 2.12. Western Blot Analysis

Cell or nuclear extracts were collected using RIPA buffer and separated on 4−12% SDS-PAGE gels followed by the transfer to PVDF membrane. The membranes were blocked with 5% dry milk and incubated with the appropriate primary antibodies. Antibodies used include iNOS, phosphor-Erk1/2, ERK1/2, phospho-JNK1/2, JNK1/2, phosphor-p38, p38, phosphor-JAK2, JAK2, phosphor-STAT3, STAT3, and GAPDH.

### 2.13. Statistical Analysis

Data were reported as the mean ± standard deviation for each individual experiment. Statistical analysis was performed using the Student's* t*-test. *P* < 0.05 was considered as significant.

## 3. Results

### 3.1. iNOS Inhibition as a Function-Guided Screen of TFRG Fractions

To determine the anti-inflammatory effect of RG fraction, we adopted iNOS inhibition as a function-guided screen of TFRG fractions. The RAW264.7 cells were pretreated with five fractions. Then, LPS and IFN-*γ* were applied for iNOS induction. Griess reaction assay showed that ethanol extract (EE), the petroleum ether (PEF), ethyl acetate (EEF),* n*-butanol (BF), and aqueous fraction (AF) of RG were all able to reduce nitrite accumulation and the IC_50_ of each fraction was 109.57, 44.40, 20.66, 100.13, and 71.72 *μ*g/ml, respectively. Because EEF possessed the strongest inhibitory effect on NO production, we further prepared TFRG from the EEF and tested its effect on NO production. Griess reaction assay showed that TFRG exhibited even stronger inhibitory effect on NO production than EEF and its IC_50_ was 7.9 *μ*g/ml (Table 1).

**Figure 1:**
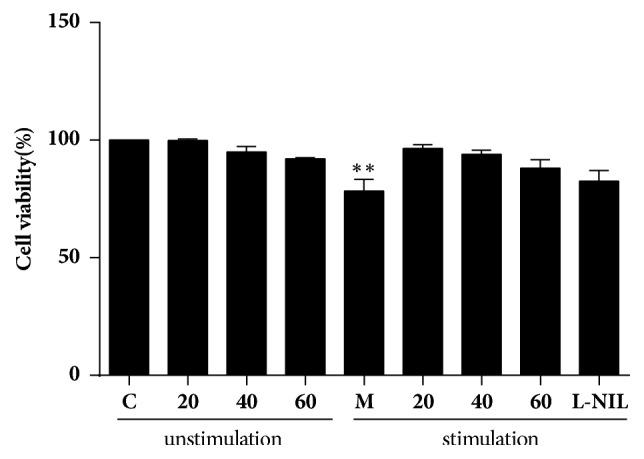
*Effect of TFRG on cell viability in LPS/IFNγ-stimulated and unstimulated RAW264.7 cells*. RAW264.7 cells were treated with TFRG (20, 40, 60 *μ*g/ml) for 24 h with or without LPS/IFN*γ* stimulation. Changes in survival are represented as percentages of the control group. C: control (nontreatment) group; M: LPS/IFN*γ*-stimulated model group; 20, 40, 60, L-NIL: treatment with TFRG (20, 40, 60 *μ*g/ml) or L-NIL (50 *μ*M) with/without LPS/IFN*γ* stimulation. Bars represent the mean ± SEM. Three independent experiments were performed. ^*∗∗*^*P* < 0.01 compared with the control group.

We next evaluated the effect of TFRG on cell viability by treating LPS/IFN-*γ*-stimulated or unstimulated RAW264.7 cells with various concentrations of TFRG for 24 h. MTT assay showed that TFRG had no apparent cytotoxicity to the unstimulated cells; meanwhile it exhibited moderate protective effect on stimulated cells since the inflammatory stimulation caused NO-depended cell damage (Figure 1). These results suggested that TFRG can safely block NO production in stimulated macrophages.

### 3.2. TFRG Suppress iNOS Expression at mRNA and Protein Level

**Figure 2:**
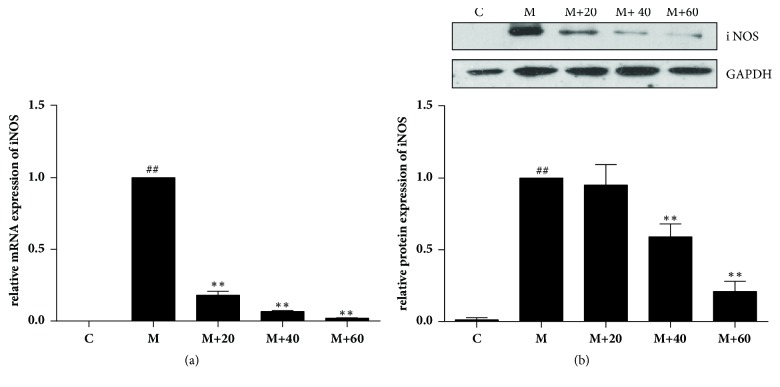
*Effect of TFRG on iNOS expression in LPS/IFNγ-stimulated RAW264.7 cells*. (a) RAW 264.7 cells (1 × 10^6^ cells/dish) were pretreated with varying concentrations of TFRG for 1 h followed by LPS (100 ng/ml) and IFN-*γ* (10 U/ml) treatment for 4 h. Total RNA was isolated and subjected to qRT-PCR. GAPDH mRNA was used as an internal control for standardization. (b) Cells were plated at a density of 1 × 10^6^ cells/well in 30-mm dishes and allowed to attach overnight. TFRG (20, 40, 60 *μ*g/ml) was added 1 h prior to the treatment of IFN*γ* (10 U/ml) plus LPS (100 ng/ml) for 6 h. Whole cells lysates were analyzed and standardized by protein concentration. The data shown are representative of three independent experiments. ^##^*P* < 0.01 versus control group; ^*∗∗*^*P* < 0.01 versus model group.

Since overexpression of NO is largely due to the elevated iNOS expression upon inflammatory stimuli, we examined the effect of TFRG on iNOS expression in RAW264.7 cells. Stimulation of LPS/IFN-*γ* induced a robust upregulation of both iNOS mRNA and protein; however, such upregulation was diminished by the pretreatment of TFRG in a dose-depended manner (Figures 2(a) and 2(b)).

### 3.3. TFRG Block LPS/IFN-*γ*-Induced NF-*κ*B, ERK Activation, and miR-155 Expression

**Figure 3:**
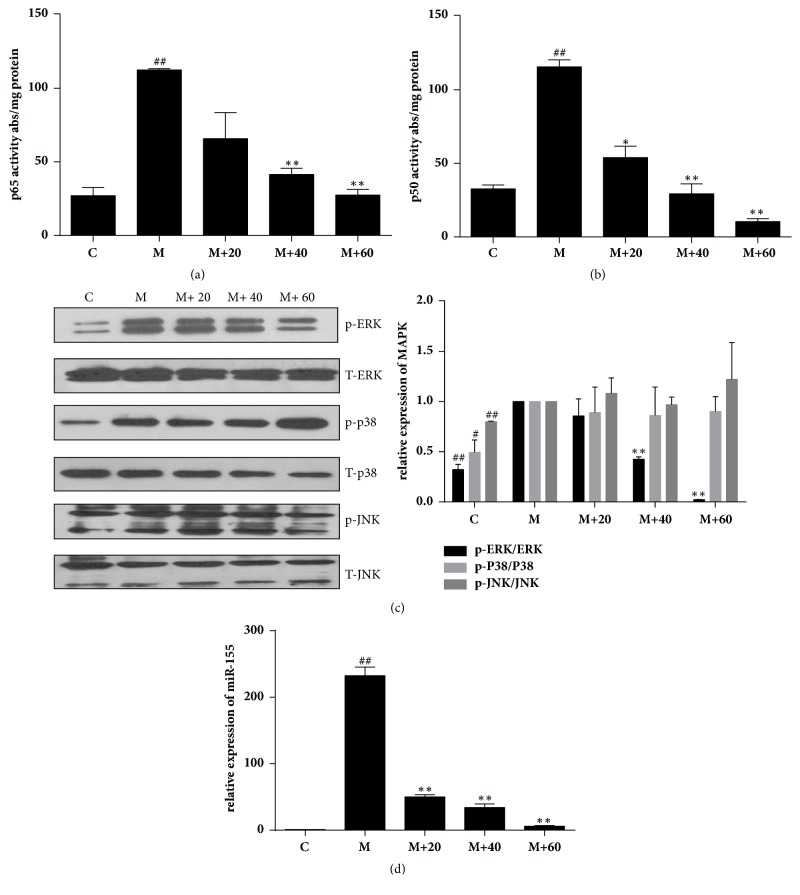
*Effect of TFRG on NF-κB, MAPK activities, and microRNA-155 expression in LPS/IFNγ-stimulated RAW264.7 cells*. (a, b) DNA binding activity of p50 and p65 proteins in nuclear extracts was assessed using NF-*κ*Bp50/p65 EZ-TFA transcription factor assay. Absorbance was measured at 450 nm in a microplate spectrophotometer. Results were normalized to absorbance/mg protein. (c) RAW264.7 cells were plated at a density of 1 × 10^6^ cells/well in 30-mm dish overnight. TFRG were added to cells for 1 h followed by 30-min stimulation of IFN*γ* (10 U/ml) plus LPS (100 ng/ml). (d) Effect of TFRG on expression of miRNA-155 in stimulated RAW264.7 cells. The cells were stimulated with LPS plus IFN-*γ* only or stimulated with different concentrations (20, 40, 60 *μ*g/ml) of TFRG for 18 h. Total RNA was isolated and the expression of miRNA-155 was determined by qPCR. RNU6B was used here as an endogenous control. The data represent the mean ± SEM of triplicate experiments. ^*∗*^*P* < 0.05 and ^*∗∗*^*P* < 0.01 compared with the model group alone.

NF-*κ*B functional pathway plays a key role in regulating iNOS expression and acts as an essential mediator in inflammatory responses [21]. To determine the effect of TFRG on NF-*κ*B activation under inflammatory condition, we assessed the amount of p50 (NF-*κ*B1) and p65 (RelA) that can bind oligonucleotides containing NF-*κ*B consensus sequence in RAW264.7 cells with or without LPS/IFN-*γ* stimulation. While only marginal binding of p50 or p65 to NF-*κ*B consensus sequence-containing oligonucleotides was observed in unstimulated cells, a dramatic increase in the amount of bound p50 or p65 was detected in stimulated cells. However, TFRG dose-dependently repressed the amount of bound p50 and p65 in stimulated cells (Figures 3(a) and 3(b)), suggesting that TFRG can interfere with NF-*κ*B signaling pathway.

Recent studies have also revealed the involvement of MAPK signaling pathways in iNOS expression [22]. To determine the effect of TFRG on MAPK activities, we treated RAW264.7 cells with TFRG followed by LPS/IFN-*γ* stimulation. Based on the extent of MAPK phosphorylation as the indicator of MAPK activity, Western blot analysis showed that LPS/IFN-*γ* led to a significant activation in ERK, p38, and JNK. Interestingly, pretreatment of TFRG partially blocked LPS/IFN-*γ*-induced phosphorylation of ERK but not p38 (Figure 3(c)), suggesting that TFRG specifically inhibit ERK signaling pathway.

Accumulated evidences demonstrated that miR-155 is involved in inflammatory responses and natural products can regulate inflammation via this multifunction miR-155 [23]. To determine the effect of TFRG on miR-155 under the stimulation of LPS/IFN*γ* in RAW264.7 cells, we pretreated cells with TFRG prior to stimulation. TFRG notably counteracted LPS/IFN-*γ* induced increase in miR-155 in RAW264.7 cells (Figure 3(d)).

### 3.4. TFRG Inhibit Tumorigenesis of MDA-MB-231 Cells

**Figure 4:**
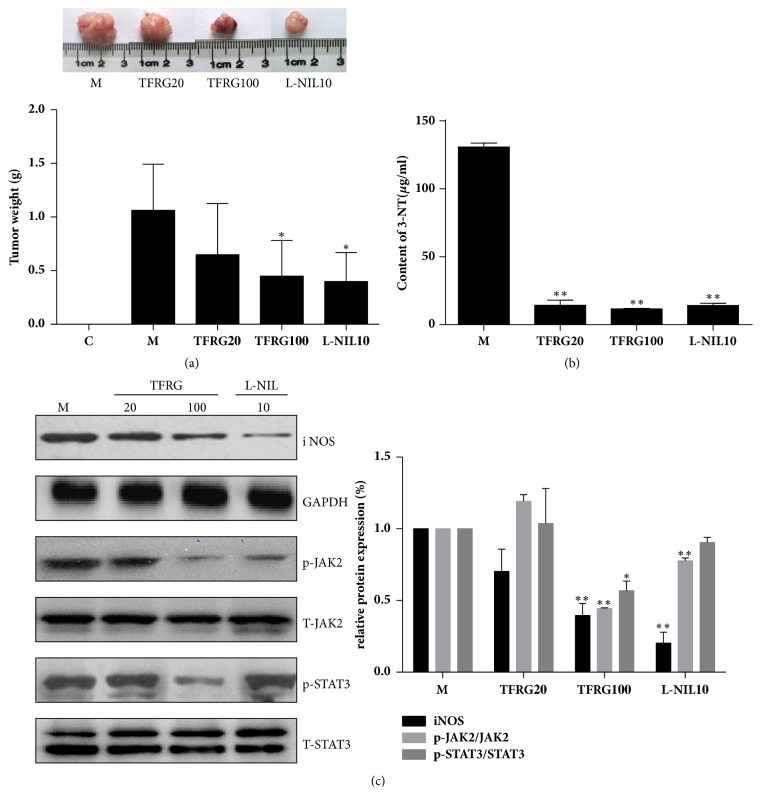
The effect on TFRG on tumor growth* in vivo*. (a) Tumors were collected and weighted. Quantification of tumor weight in vehicle, TFRG (20, 100 mg/kg/d) with indicated concentrations and L-NIL (10 mg/kg/d), the positive control groups. ^*∗∗*^*P* < 0.01, ^*∗*^*P* < 0.05 versus model group. (b) The tumor tissues with/without TFRG and L-NIL were excised quickly and stored in −80°C. Detection content of 3-NT was carried out according to the kit protocol and data were quantified with mg protein of each sample. (c) The tumors of mice treated with/without TFRG and L-NIL were collected and subjected to Western blotting to detect iNOS, phosphor-JAK2/total JAK2, and phosphor-STAT3/total STAT3. M: model group; TFRG 20, TFRG 100: TFRG at 20, 100 mg/kg/d; L-NIL10: L-NIL at 10 mg/kg/d dosage.

We investigated the effect of TFRG on tumor outgrowth by establishing breast tumor orthotopic xenograft model through the injection of MDA-MB-231 cells in female nude athymic nude mice at mammary fat pads. Two weeks after cells injection, animals were either administrated with or without TFRG for 4 weeks. We also included groups in which mice received L-NIL, a specific iNOS inhibitor, respectively, for the purpose of validating the link of iNOS between inflammation and tumor progression. At the end of treatment period, mice were sacrificed and tumors were excised. Treatment of TFRG suppressed over 50% of the tumor weight (Figure 4(a)). L-NIL also suppressed tumor outgrowth. Our results support the notion that tumor development can be suppressed through the inhibition of inflammation.

### 3.5. TFRG Suppress iNOS and 3-NT by JAK2/STAT3 Pathway

Given the importance of iNOS in breast cancer development, we reasoned that eradicating the production of iNOS will lead to the suppression of tumor progression. In subsequent experiments, we examined the effect of TFRG on iNOS and 3-NT in the tumors of MDA-MB-231 xenografts. We showed that TFRG significantly decreased the level of iNOS and JAK2/STAT3 in tumors (Figure 4(b)). The content of 3-NT was much less in the xenografts derived from mice receiving TFRG compared to mice receiving only vehicle (Figure 4(c)). Similarly, L-NIL was also able to attenuate the formation of iNOS and 3-NT, confirming the role of iNOS driven-inflammation in breast cancer development. These results demonstrate that TFRG are potent inhibitors of iNOS-associated inflammatory responses during breast cancer growth.

### 3.6. Quality Control of TFRG by UPLC Analysis

**Figure 5:**
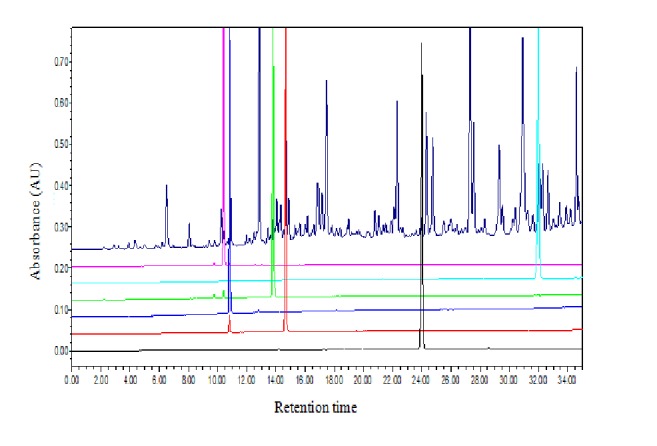
*High performance liquid chromatography profiles at 254 nm of TFRG*. From the top to the bottom: (1) TFRG, (2) Isoliquiritin (pink), (3) Isoliquiritinapioside (light blue), (4) Glycyrrhetinic acid (green), (5) Liquiritinapioside (blue), (6) Isoliquiritigenin (red), (7) liquiritin (blank).

According to the regression equation between standard quantity (*X*) and absorbance (*Y*) using liquiritin as standard (*Y* = 0.4938,* X* = 0.0003, *R*^2^ = 0.9999), US results showed that the content of total flavonoids in TFRG was 58.11%. Among TFRG, the contents of Isoliquiritin, Isoliquiritinapioside, Glycyrrhetinic acid, Liquiritinapioside, Isoliquiritigenin, Liquiritin were 2.1, 0.52, 1.34, 0.5, 0.3, and 5.14% respectively (Figure 5).

## 4. Discussion

According to Chinese Traditional Medicine (TCM) theory, the root of RG, which commonly called licorice, is sweet in flavors, moderate in natures, and relates to spleen, stomach, heart, and lung in meridian distributions. Its medicinal functions include nourishing spleen and enriching Qi, damping lung to remove cough, weakening poisonous effect of some herbs, and coordinating the interactions among various herbs in formulas [24].

Flavonoids are abundant in plants, in which they perform several functions. They are essential pigments for producing the colors needed to attract pollinating insects. In higher order plants, flavonoids are also required for UV filtration, nitrogen fixation, and cell cycle inhibition and as chemical messengers. Flavonoids are ubiquitous in plants and are the most common type of polyphenolic compound found in the human diet [25]. The flavonoids in RG possess diverse biological/therapeutic effects including neuron-protective [26, 27], anti-insulin resistance [28], gastrointestinal function-regulatory, and analgesic. In terms of antitumor properties of these flavonoids in RG, the effects of proapoptotic [29–31] and antimetastatic [32] properties have been reported in various cancer models and/or cell lines.

Reactive oxygen and nitrogen species (ROS and RNS) and iNOS, which can be induced through NF-*κ*B and MAPK signaling pathways, are important inflammatory mediators that can facilitate tumor growth, metastasis, and immune evasion/survival [33, 34]. Because of the causative roles of these factors in carcinogenesis, they have been considered as potential therapeutic targets [35, 36]. Chronic inflammation is recognized as one of cancer hallmarks [37, 38]. The relationship between inflammation and cancer is supported by the observation that prolonged use of nonsteroidal anti-inflammatory drugs (NSAIDs) is associated with reduced risk to developing various types of cancers [39]. With the setting of murine macrophage RAW264.7 cells experimental system, we showed that TFRG from RG effectively blocked LPS/IFN-*γ*-induced NO production and iNOS expression without cytotoxicity (Table 1; Figures 1 and 2). We further showed that TFRG exerted their anti-inflammatory effect by interfering with NF-*κ*B and ERK/MAPK signaling pathways and miR-155 expression (Figure 3).

Given the potent anti-inflammatory effect of TFRG observed in our experimental model, we hypothesize that they should also possess antitumorigenic effect. We showed that TFRG were able to downregulate the levels of iNOS via JAK2/STAT3 signal pathway which were highly expressed and activated in tumor tissues (Figure 4). Free or protein-bound tyrosines are constantly converted to free or protein-bound 3-NT through the attack by various RNS including peroxynitrite. The abundance of 3-NT has been used as an indicator for RNS conditions [40]. We showed that the amount of 3-NT in the tumor xenografts notably decreased by TFRG treatment (Figure 4). Our speculation is actually backed by the observation that iNOS inhibitor L-NIL was also capable of effectively blocking tumorigenesis of MDA-MB-231 cells. In additional, we identified some constitutes conforming TFRG using US-UPLC (Figure 5).

**Figure 6:**
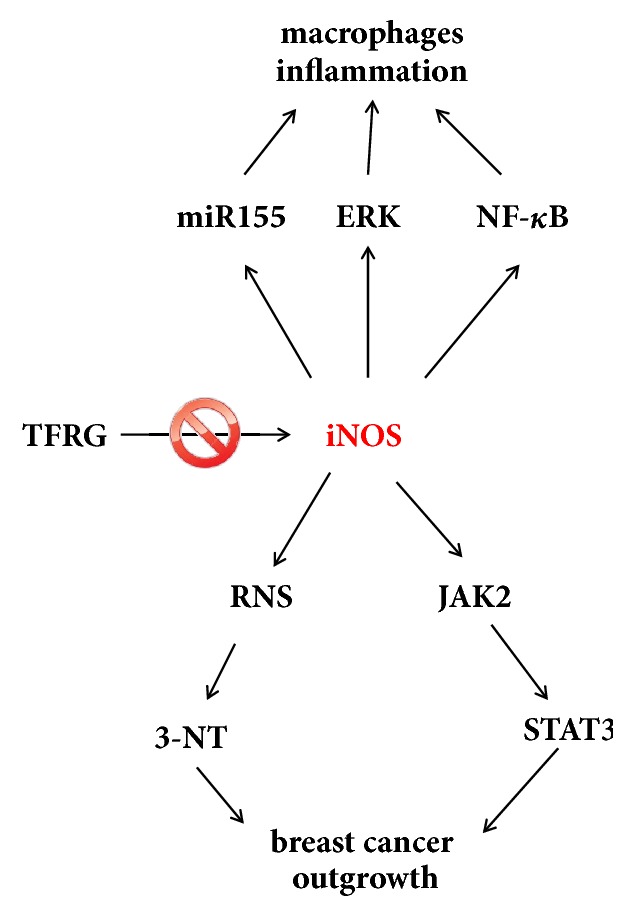
The potential mechanisms of TFRG in suppression of breast cancer and inflammation.

In conclusion, the data presented indicates that iNOS can be induced under inflammatory and tumorigenic conditions, and TFRG as well as iNOS inhibitor L-NIL diminished iNOS driven responses in inflammation and tumorigenesis (Figure 6).

## Figures and Tables

**Table 1:** *Effect of the ethanol extract and fractions from Radix Glycyrrhiza on NO production in LPS/IFNγ-stimulated RAW264.7 cells*. RAW264.7 cells were treated with extract (10, 100, 200 *μ*g/ml), each fraction (10, 100, 200 *μ*g/ml), and TFRG (20, 40, 60 *μ*g/ml) for 24 h with LPS/IFN*γ* stimulation. Nitrite accumulation in the culture medium was determined by the Griess reaction. C: control (nontreatment) group; M: LPS/IFN*γ*-stimulated model group; EAA: ethanol extract with stimulation; PEF: petroleum ether extracted fraction with stimulation; EEF: ethyl acetate extracted fraction with stimulation. BF: *n*-butanol extracted fraction with stimulation; AF: aqueous extracted fraction with stimulation; TFRG: total flavonoids of RG with stimulation; L-NIL: L-NIL at 50 *μ*M with stimulation. Values are presented as the mean ± SEM from three replicates. ^##^*P* < 0.01 versus C group;^*∗*^*P* < 0.05 versus M group;^*∗∗*^*P* < 0.01 versus M group.

Group	Extracts/fractions	Dose (*μ*g/ml)	Nitrite (*μ*M)	Inhibition (%)	IC_50_ (*μ*g/ml)	Yield (%)
C	-	-	1.28 ± 0.01			
M	-	-	64.65 ± 0.02^##^			
RG	Ethanol extract (EE)	10	66.15 ± 0.04	−2.36	109.57	31.6
		50	43.25 ± 0.04^*∗∗*^	33.77		
		100	35.85 ± 0.03^*∗∗*^	45.46		
		200	19.85 ± 0.02^*∗∗*^	70.7		
	Petroleum ether fraction (PEP)	10	56.62 ± 0.02^*∗∗*^	12.63	44.4	7.02
		50	40.09 ± 0.01^*∗∗*^	38.76		
		100	17.20 ± 0.02^*∗∗*^	74.88		
		200	5.16 ± 0.01^*∗∗*^	93.9		
	Ethyl acetate fraction	10	52.41 ± 0.03^*∗∗*^	19.31	20.66	11.05
	(EAF)	50	12.05 ± 0.02^*∗∗*^	83.02		
		100	3.39 ± 0.01^*∗∗*^	96.68		
		200	3.26 ± 0.01^*∗∗*^	96.89		
	*n*-Butanol fraction (BF)	10	54.15 ± 0.03	−0.2	100.13	12.56
		50	40.27 ± 0.03^*∗∗*^	26.13		
		100	32.26 ± 0.01^*∗∗*^	41.32		
		200	7.54 ± 0.03^*∗∗*^	88.19		
	Water fraction (WF)	10	59.40 ± 0.02^*∗∗*^	8.29	71.72	26.39
		50	56.98 ± 0.03^*∗∗*^	12.11		
		100	28.05 ± 0.01^*∗∗*^	57.76		
		200	5.93 ± 0.01^*∗∗*^	92.68		
	TFRG	20	11.46 ± 0.02^*∗∗*^	77.67	7.9	1.95
		40	5.76 ± 0.01^*∗∗*^	93.89		
		60	5.67 ± 0.01^*∗∗*^	94.16		
	L-NIL	50 (*μ*M)	32.74 ± 0.03^*∗∗*^	30.2		

## Data Availability

All the data supporting the results in this manuscript can be found.

## Abbreviations

RG: Radix Glycyrrhiza
TFRG: Total flavonoids of Radix Glycyrrhiza
LPS: Lipopolysaccharide
IFN-*γ*: Interferon-*γ*
NO: Nitric oxide
iNOS: Inducible nitric oxide synthase
RNS: Reactive nitrogen species
3-NT: 3-Nitrotyrosine
NF-*κ*B: Nuclear factor-kappa B
ERK: Extracellular signal-regulated kinase
MAPK: Mitogen-activated protein kinase
JNK: c-Jun N-terminal kinase
US: Ultraviolet spectrophotometry
UPLC: Ultraperformance liquid chromatography
TME: Tumor microenvironment
TCM: Traditional Chinese Medicine.
